# A Biocratic Paradigm: Exploring the Complexity of Trauma-Informed Leadership and *Creating Presence*™

**DOI:** 10.3390/bs13050355

**Published:** 2023-04-24

**Authors:** Sandra L. Bloom

**Affiliations:** Dornsife School of Public Health, Drexel University, Philadelphia, PA 19104, USA; slb79@drexel.edu

**Keywords:** trauma-informed, traumatogenic, trauma-organized systems, stress, biocracy, leadership, *Creating Presence*™, worldview, paradigm shift, adverse childhood experiences

## Abstract

A paradigm shift is under way in the human services because of breakthrough knowledge and research in understanding the underlying etiology of physical, emotional, and social problems at the micro-level of the individual, at the meso-level of the family and institutions, and at the macro-level of the entire society. The three levels of human existence—micro, mezzo, and macro—constitute interactive, interdependent, complex adaptive living systems. The complexity of these problems requires us to use our imaginations to envision health in individuals, organizations, and societies because it does not presently exist. After thousands of years of unrelenting exposure to trauma and adversity, we have all normalized what is a traumatogenic civilization. As a result, we live in a trauma-organized society in ways we are just beginning to understand in this century. This biopsychosocial knowledge base that is drawn upon here has come to be known as “trauma-informed” knowledge because it began with a deepening understanding of the impact of trauma on survivors of combat, disasters, and genocide, but now extends far beyond those specific boundaries. To lead any organization in a time of significant change means leading a revolution in understanding human nature and the fundamental causes of human pathology that are endangering all life on this planet and then helping organizational members develop skills to positively influence the changes necessary. In the 1930s, Dr. Walter B. Cannon, a Harvard physiologist who had named the “fight-flight” response and defined homeostasis, used the word “biocracy” to describe the relationship between the physical body and the social body, emphasizing the vital importance of democracy. This paper is a beginning attempt at integrating the concept of a biocratic organization with that of the trauma-informed knowledge necessary for leadership. Hope lies in properly diagnosing the problem, remembering ancient peace-making strategies, embracing universal life-preserving values, inspiring a new vision for the future, and radically and consciously changing our present self and other-destructive behavior. The paper concludes with a brief description of a new online educational program called *Creating Presence*™ that is being used in organizations as a method for creating and supporting the development of biocratic, trauma-informed organizations.

## 1. Introduction: Shifting Paradigms

Shifts in worldviews are called “paradigm shifts”. A paradigm represents society’s deepest set of beliefs about how the world works. Paradigms are the source of systems, so a paradigm shift means transformation, revolution, or metamorphosis and those kinds of change frighten people as if the Earth were quaking right under their feet. In an organization it means that everyone needs to change by acquiring knowledge and skills that lead to changes in practice and policy. The last big shift in medicine was the discovery that microbes were the cause of infectious disease and as a result many of us are alive today who otherwise would not have been. Scientific paradigms change mental models, which represent the basic understanding we have about the rules for how the world works, and as our mental models change, we experience changes in attitudes and ultimately changes in the way we behave [1].

The current paradigm shift that began with quantum physics has taken a century to begin to influence how we think about human beings and human systems. Clocks work as clocks do, but people do not work like clocks and therein lies the challenge for leading anyone, whether that means parenting children, running a hospital, or leading a multibillion-dollar enterprise. Thomas Kuhn described the stages of a paradigm shift occurring when “normal science” is confronted with increasing numbers of “anomalies” until there exist in the world two radically different ways of understanding and addressing problems—two very different worldviews. When the new paradigm solves more previously unsolvable problems than the old, it becomes the new “normal” science [2]. That helps to explain why our world currently is profoundly divided—there are two very different worldviews dominating the environment.

Over the last fifty years, the multigenerational impact of childhood adversity and trauma has become abundantly evident, so much so that in this paper the claim is that we are living in a civilization that is “traumatogenic” in ways that have been largely ignored in our understanding and explanations of human motivations and behaviors, and that may be understood as root causes of adult mental illness, addiction, and medical disease [3,4,5,6] As a result, our systems and organizations have become “trauma-organized” in ways that are difficult to understand without a thorough knowledge of the impact of adversity and trauma, a knowledge base now called “trauma-informed” [7].

One of the most important physiologists of the first half of the 20th century, Dr. Walter B. Cannon, the same scholar who initially described the “fight-flight” response and studied “homeostasis”, laid down important groundwork that this paper asserts can guide our thinking in a way that can help us to deliberately and consciously restructure our currently dysfunctional workplaces that are having such an enormous impact on burnout, parenting, and our interpersonal lives [8,9]. This paper elaborates on his original ideas for “biocratic” organizations, living systems modeled after the human body, that may guide us in formulating strategies that support organizational health instead of tolerating dysfunction [10,11,12].

Becoming “trauma-informed” means changing one’s own worldview of how and why the world works as it does and this requires a major intrapsychic adjustment around common everyday assumptions that we have been taught and that are reinforced every day within the organizations and institutions we interact with—our own mental models. Only in changing our mental models can we expect to achieve changes in attitudes and ultimately in behavior. Becoming a trauma-informed leader necessitates deep level change you will need to make room for in your organization and, because these are still very early days in this profound paradigm shift in understanding and responding to human nature, you most definitely will encounter resistance to change in yourself and in everyone you encounter.

Based on decades of experience in first treating people suffering from the complex disorders that arise secondary to adversity and trauma, and then several more decades of helping organizations to become trauma-informed, this paper introduces and summarizes a new online organizational approach we are calling *Creating Presence*™ that can assist whole organizations in making the paradigm shift that is being described in this paper and join a community of practice comprised of many different kinds of workplaces who are endeavoring to become trauma-informed.

Since our physical evolution has left us with one foot in the 21st century and the other solidly planted in the Stone Age, ultimately this shift in paradigm will require a leap in social evolution [13]. The knowledge we have gained in the last century about attachment, stress, adversity, and trauma all contribute to a very different paradigm for understanding human nature and the institutions we humans create. The question is, can we make use of this knowledge before it is too late? In the remaining pages, imagine a different way of being in the world, of being a leader of a “biocratic” system.

## 2. The Science of Suffering

Beginning in the 1980s, my colleagues and I developed some ideas about what constitutes “trauma-informed leadership” by first creating and then leading an inpatient psychiatric program and a large outpatient practice for two decades that specialized in the treatment of adult survivors of child maltreatment and other forms of trauma. We then had the opportunity to share those lessons learned with several hundred other organizations in the United States and in various other places around the world [7,14,15,16]. This is how what I have called the “science of suffering” became the center of our lives. Now we are developing and applying an online training program called *Creating Presence*™ to help even more organizations participate in the paradigm shift.

As a current or aspiring leader, you too have a responsibility to grapple with the many personal and organizational implications of this science of suffering, the extensive body of research that has been accumulating gradually during the 20th century but that accelerated rapidly after the Vietnam War [17]. This body of both quantitative and qualitative research is now sufficient in magnitude to comprise the scientific biopsychosocial and genetic underpinning for a significant shift in paradigm in our understanding of—and potentially prevention of—child abuse, domestic violence, multiple chronic health problems, mental disorders, criminality, substance abuse, poverty, warfare, moral injury, and moral disengagement. This science records the unrelenting suffering that man has imposed on men, women, children, and all living things across at least the last several thousand years [18,19,20,21].

### 2.1. The Problem with Words

We do not have a word that accurately represents the enormity of the impact of overwhelming life events on human beings, so we use the word “trauma” to stand in for a complexity that represents a significant shift in our understanding about the nature of human-experienced reality in the present and in the past. Knowledge begins with understanding the many manifestations of stress. Stress is the nonspecific response of the body and brain to any demand and represents the normal wear-and-tear of daily existence [22]. There are positive forms of stress and negative forms of stress and the latter can be broken down into relentless stress, toxic stress, and traumatic stress that occur in the lives of individuals, organizations, communities, and whole societies in the forms of collective, cultural, and historical trauma [23,24,25].

### 2.2. Childhood Suffering

Research has shown that for most people around the world, human suffering begins in infancy and early childhood and is almost universal, although universally denied. Decades of research have consistently linked physical punishment with risks of harm to children and yet over 80% of American parents have assaulted their children [26,27,28,29,30]. A quarter of a century of research studying the impact of adversity and trauma in childhood is truly staggering in its implications since we now know that toxic stress—the excessive or prolonged activation of stress response systems in the body and brain during critical development periods—has damaging effects on learning, behavior, and health across the lifespan and this is happening to children around the world [3,31,32].

Now, why would childhood adversity be so damaging? Largely because it creates attachment trauma for the child during critical developmental periods of the brain and body [31,33]. *Anything* that interferes with the loving, caring relationship between mother and child during the fetal period and during the first months and years of life damages the child’s brain in ways we are just learning about [34]. Some reorganization can later occur in adolescence because of the influence of the sex hormones and that is what makes adolescence so vitally important as well. That *anything* includes physical and/or sexual violence against women which even today happens to more than 1 out of 4 women worldwide at the hands of male intimate partners beginning when women are 15 years old [35].

### 2.3. Traumatogenic Culture

*Every region has its own distinct trauma signature. It’s as if a massive elephant sits in the human living room; few may see or acknowledge it, but we are all impacted by its presence. Everything about our societies—from geopolitics to business, climate, technology, health care, entertainment and celebrity, and much more—is dominated by the existence of this elephant, by the residue of our collective trauma. And as long as we fail to acknowledge or adequately care for it, the elephant will grow larger* (p. 22) [19].

Thomas Hübl, 2020, *Healing Collective Trauma*.

We all live in cultures that create, participate in, and permit the creation of traumatic, toxic, and relentlessly stressful lives for almost all of us beginning in childhood. Many years ago, borrowing a term from dentistry, I coined the term “traumatogenic” to apply to social constructions in our society that have become backdrops against which the chances of exposure to violent perpetration and violence as a problem-solver are increased [36]. This idea of the pathology of cultural communities, as Freud described it, has yet to be fully explored but it is not a new idea—although it is a concept that keeps being denied and therefore is repeatedly lost to human consciousness [37,38,39].

Besides childhood adversity, we also know that other types of traumatic events are highly prevalent worldwide, with studies across a broad range of countries estimating that between 70% and 90% of adults have suffered at least one traumatic event during their lifetime, and 30.5% have been exposed to four or more [40,41]. According to the National Center for PTSD, most Americans will experience a traumatic event. Women are more likely to experience sexual assault and child sexual abuse, both known to lead to higher rates of post-traumatic stress disorder than many other kinds of trauma. Men are more likely to experience accidents, physical assault, combat, disaster, or to witness death or injury [42]. In addition to the multiple medical complications associated with trauma, exposure to psychological trauma is associated with nearly three times greater risk of having a mental disorder—any mental disorder—and the authors of this “umbrella review” concluded that psychological trauma is what they termed a “transdiagnostic” risk factor for mental disorder [43].

Space does not allow the exploration of many other social problems: poverty, racism, sexism, exploitation of workers, pornography, substance abuse, media violence, injustice, incarcerations, vast inequality, natural and manmade disasters, etc.; however, all of these problems combined produce conditions that are increasingly adverse for childrearing and that radically alter our social norms. Hence, whatever damage has occurred in one generation is likely to manifest in parenting of the next generation not only at a behavioral level but also at an epigenetic level as disrupted attachment leads to epigenetic changes that continue to influence parenting behavior down through the generations [44].

It is impossible to understand what has gone so wrong for the human species unless you understand attachment trauma and thousands of years of disrupted attachment as a result of adversity and trauma and the pathological belief systems that have arisen as a result [39]. The combined effect of all of these factors is to create a sense of existential confusion and a profound questioning of purpose and meaning that so characterizes the social environment of the late 20th century and the early decades of the 21st [36]. One noted philosopher has gone so far as to make the case that human evil is a form of pathology that has become the normal state of humanity [38].

### 2.4. Trauma-Organized Systems

*One way of helping people is by reminding them that the time is getting late, that the situation is grave, that it can’t be ignored. Seeing the outlines of horror induces the will to face up to it* (p. 199) [45].

Vaclav Havel, 1990, *Disturbing the Peace*.

As you assume a leadership position it is likely that you are walking into a situation that has existed for some time already and has a history that you may or may not know anything about. That means you will need to understand the concept of parallel process. A historical perspective means that what we are now is a product of thousands of years of exposure to natural and manmade trauma. Parallel process is a longstanding term familiar to many people in social service settings wherein “*two or more systems—whether these consist of individuals, groups, or organizations—have significant relationships with one another, they tend to develop similar thoughts, feelings and behaviors*” (p. 13) [46]. The concept of parallel process derives from the psychoanalytic concept of transference between therapist and client, comprising largely unconscious processes that need to be surfaced in the supervisory relationship, but similar processes are characteristic of all complex adaptive living systems. The researchers quoted above go on to state that “*Parallel processes can be set in motion in many ways, and once initiated leave no one immune from their influence. They can move from one level of a system to another, changing form along the way*” (p. 13) [38]. What is emerging are underlying laws that can be applied to systems in general and that are therefore applicable across all disciplines [47]. To the extent this is true, conflicts and tension within interactive and interdependent systems are likely to produce rippling effects in other connected systems [48]. The recent COVID-19 pandemic has illustrated that impact in ways that many people were largely unaware of until those parallel effects hit them as individuals, as families, as employees, as employers, as institutions, and as governments.

A traumatogenic culture produces *trauma-organized* individuals, families, organizations, and communities that become stuck in time, repetitively adapting to circumstances and events that have already happened, but not responding well to the present and creating a predictable but highly problematic future. I have defined a *trauma-organized system* as one that is fundamentally and unconsciously organized around the impact of chronic and toxic stress, even when this undermines the essential mission of the system [14,49,50,51]. The more people there are in an environment with unrecognized, untreated, chronic, or continuous exposure to the stressful conditions that have been and continue to be a fundamental part of our environments, the more likely that parallel processes of dysfunction will occur and affect everyone—top to bottom.

## 3. The Genius of Dr. Walter B. Cannon: Biocracy

Dr. Walter B. Cannon, considered to be one of the most important scholars of the 20th century, was a Harvard physiologist who coined the term “fight or flight” and defined the concept of “homeostasis” or balance that defines most of the function of the living body. He understood that our socially constructed systems are alive, just as the human body is alive. In 1936 he wrote, “*it seems to me that quite possibly there are general principles of organization that may be quite as true of the body politic as they are of the body biologic*” (p. 206) [10]. In 1940 he became President of the American Association for the Advancement of Science and in his Presidential Address he asserted that the most efficient and stable human society would be a “*biocracy in which the myriad of differentiated cells would be organized into functional organs all cooperating in a dynamic democracy in which any form of dictatorship would lead to degeneration and death*” (p. 1) [52].

Dr. Cannon’s ideas about biocracy have received some notice across the past eighty years. “Organicism”—the notion that the social body is analogically identical to the physical body—has deep roots in the thinking of Comte, Spencer, and Durkheim [53]. Caldwell was probably the first relatively recent author to connect biology, democracy, science, ethics, and the law in a coherent political theory with applications to public policy [54,55]. At least one author has raised important questions about the application of biocratic principles to the workplace including whether or not it potentially makes us more free or more constrained and exploited [56,57]. Other authors connect the concept of biocracy to the rights of nature, to climate change, and to a different approach to cities [58,59,60]. A retired microbiologist, W.T. Martin, has written a book describing biocracy as “democracy that includes nature”. However, as far as I am aware, this paper is the first attempt to integrate the ideas around biocratic organizations with the study of trauma and adversity.

Thanks to Dr. Cannon, we can launch a very different way of looking at all our systems in parallel—individual, organizational, and societal. All of us have at least a rudimentary idea about how our bodies work. Therefore, the human body can serve as a good model for some of the characteristics of other living, complex adaptive systems. A living complex adaptive system—every individual, organization, community, and society—has many characteristics that are very different from machines, among them unpredictability, openness to new information and learning, memory, and a capacity for self-organization. At the same time, living systems have something called “sensitive dependence on early conditions”—or in human terms, “childhood”—and yet remain very adaptable and able to repair injury. Perhaps most importantly, living systems have *choice* within whatever biological constraints exist for that living system [61,62,63]. All of this is relevant information that a trauma-informed leader must understand if he, she, or they are to guide a biocratic institution.

### 3.1. Healthy Organizations Are Made Up of Healthy People: What to Look for in Individual Leaders and Employees

The recognition that personality health/disturbance is on a continuum has evolved over decades of clinical observation and research. A group of psychologists have taken on the challenge of defining individual health. At the healthy end of the continuum are people who show good functioning in all or most of the following domains, in which they can engage in satisfying relationships; experience and understand a relatively full range of age-expected feelings and thoughts; function relatively flexibly when stressed by external events or internal conflict; maintain a relatively coherent sense of personal identity; express impulses in a manner appropriate to the situation; conduct themselves in accordance with internalized moral values, and neither suffer undue distress nor impose it on others [64].

Of course, by this definition, there may be very few psychologically healthy individuals in our culture. Is it even possible in our culture to never impose undue distress on oneself or others? As the numbers of children, adults, families, and communities who have experienced trauma appear to be multiplying exponentially, and while funding for the provision of complex services is plummeting, there is an urgent need to find more efficient and cost-effective methods for re-educating the entire workforce in the best methods for enhancing resilience and post-traumatic growth while promoting healing and recovery. If we cannot count on having a workforce of psychologically healthy and stable individuals, then it will be necessary to do our best to create and sustain healthy workplace cultures that are able to counteract whatever damage has already been done to people at home and in the workforce and then, going forward, prevent any more damage from occurring. It must be a culture that promotes individual health as well as organizational health. One of the fundamental challenges for a trauma-informed leader is creating or altering the organizational culture in such a way that health and wellbeing are encouraged and supported.

### 3.2. Healthy Organizations Are “Biocracies”

It does not matter where you work, your workplace can be defined as a “complex adaptive system”—a living system. This is why so many people work within problematic and dysfunctional workplaces. For the most part, healthy organizational cultures do not exist. The traumatogenic factors in Western societies have become so potent that no one is left unaffected by normative behaviors that create adversity for others, what Erich Fromm years ago termed the “*pathology of normalcy*” [65]. He very clearly expressed this pathology when he noted that “*destructiveness and cruelty…constitute a paradox: they express life turning against itself.... They are the only true perversion. Understanding them does not mean condoning them. But unless we understand them, we have no way to recognize how they may be reduced, and what factors tend to increase them*” (p. 9) [66].

Living, vital organizations are not machines but all too often we act as if they function mechanically and that alone makes problem-solving more difficult [67,68]. However, we have not had a good definition of a healthy person, much less a healthy organization. The Center for the Developing Child at Harvard has defined health as “*more than merely the absence of disease—it is an evolving human resource that helps children and adults adapt to the challenges of everyday life, resist infections, cope with adversity, feel a sense of personal well-being, and interact with their surroundings in ways that promote successful development*” (p. 2) [69]. If we use this definition and modify it for the health of a larger social body, we need to say that organizational health is “*an evolving human resource that helps the organization and everyone who comprises it to adapt to the challenges of everyday life, resist infections, cope with adversity, feel a sense of well-being and interact with their surroundings in ways that promote successful development*”.

If they are to be healthy and thrive, living systems require food, love (broadly defined as “care”), and sufficient protection to keep them safe while not restricting development and further growth. To provide those basic requirements means that, as a leader, you must create organizational situations where there is sufficient funding, space, staffing, administration, and security. For an organization, funding is analogous to food, water, and shelter for a person. Without those basic requirements the organization cannot continue to survive. At this point in time, many of our most vital caregiving environments are dead or dying because of a chronic state of starvation with all the problems that go along with insufficient nutrition. The physical body of the organization—where it is located in space, the design and furnishing of that space—must be maintained and, in many cases, restored to a better level of fitness. A deformed, poorly maintained, run-down and ugly organizational physical body devalues all the people working within it.

The ways in which energy is distributed within the organization, and therefore the level of organizational health, depends on the essential purpose of the organization. To be a trauma-informed leader, you will need a very well-defined and clear sense of purpose and you will need to define for everyone what you want your organization to accomplish. Beginning with this defined purpose, everything that you do, every person hired, every policy created must serve that defined organizational purpose. Conflicts may arise when an organization has more than one purpose and those conflicting demands can be quite challenging. For example, you come into the organization with your key purpose being to serve the needs of traumatized young people only to recognize that another key purpose that no one openly discussed in your interview for the job, is to provide jobs for adults who are living within historically impoverished communities. In such a case it is important to surface both purposes from the start so that no matter what conflicts arise in meeting both demands, thought can be given to how to resolve such conflicts. If you fail to make the conflict conscious, it is likely that your primary mission will be sabotaged by the other mission without anyone necessarily being aware that this is precisely what is happening.

### 3.3. Biocracies Are Learning Organizations

Human beings must learn how to survive and thrive in the world from cradle to grave and organizational bodies are no different. Peter Senge has written extensively about the learning organization and predicted that “the organizations that will truly excel in the future will be the organizations that discover how to tap people’s commitment and capacity to learn at all levels in an organization” (p. 4) [55]. This means that all people within organizations must learn how to do systems thinking so that learning is distributed throughout the entire corporate body. Then, every individual must develop personal mastery and approach their work as an art, as a deeply spiritual and moral commitment that requires life-long learning [56].

Complex adaptive systems learn and use that new information to alter present and future behavior. Learning comes from experience in interaction with other parts but also from sensations and feelings that alert living systems to change and to injury—and, being alive, complex adaptive systems can be stressed, injured, and traumatized. They can die and they can be inadvertently killed or deliberately murdered, but complex adaptive systems are interdependent and interactive with every other component part of a complex adaptive system. One injured component of a complex adaptive system is not living in isolation and therefore any dysfunction or death will affect every interactive component of the entire system.

A learning organization challenges its existing mental models, especially when encountering difficult challenges to adaptation using “double loop learning” where a group can challenge its underlying basic assumptions—its mental models—and determine if it is the mental model that must change to meet the challenge [57]. The biggest challenge currently to creating and sustaining trauma-informed learning organizations is that the whole notion of trauma and adversity challenges many of our existing individual and group mental models. Changing those is no small feat. A learning organization creates a shared vision and teams get into alignment around that vision as they figure out how to achieve the organizational purpose. Let us look at two of the key words in this description: *complex* and *adaptive.* The interconnected and interactive impacts of many diverse elements is what makes a living system complex and often therefore unpredictable. At the same time, living systems must constantly adapt to changing conditions and alterations in their internal and external environments. This adaptation occurs through *feedback loops* that keep the various components of a system in communication with each other and in doing so produce the homeostatic balancing that maintains life [70]. Being a trauma-informed leader means that you must be central to guaranteeing that the homeostatic mechanisms throughout the system are operating correctly once new learning has occurred. To make that happen, you will need to decide how every component part of your organization will have the time necessary to learn and process the trauma-informed educational material that they will need to function in a healthy way, and to restore whatever impairments to health and well-being have occurred. The COVID-19 pandemic has illuminated many of the chronic problems that have only been exacerbated by this global stressor and has demonstrated to everyone the reality and impact of living complex adaptive systems under severe stress [71,72,73].

For living systems to accept and transfer information they need sense organs and emotions that help them to determine what to pay attention to and what they can safely ignore. This requires an *openness*, another of the outstanding characteristics of a living complex adaptive system, making it possible for the system to accept information from its environment and to act on the environment in return. A closed system is one in which there is no transfer of information, situations that researchers try to create, to gain some control over experiments, but often fail because of the very nature of living systems.

Over time, as learning occurs, living systems develop *identity* and *memory*. As learning and adaptation occur constantly in response to changing conditions, these memories impact the ways in which the system functions in the present and at least partially determine future adaptations. In this way the past is always wholly or partially constraining future possibilities. This has become known as *sensitive dependence on early conditions*, and helps to explain why, in human beings, childhood is so important—because the early developmental stages of human organizations and human individuals play such an important role in determining all that follows.

It has now been established that exposure to childhood adversity is almost universal. This has enormous consequences for providing trauma-informed leadership. You must be able to identify the signs of adversity in the multiple interpersonal conflicts you will inevitably need to address. That means you will need exemplary skills in conflict management, looking and finding win–win, instead of the more typical win–lose, scenarios. It is unlikely that you will always get everything right and there will be times that you inadvertently injure people—not their physical selves but instead their feelings and self-esteem. You must remember that living systems are frequently injured and have the capacity to both *adapt* and *self-repair*. To facilitate the adaptation and repair processes you will need a high level of emotional intelligence along with the confidence necessary to address the ways in which you have psychologically injured and even betrayed people who trusted you. It is important that you remember that individuals and organizations *evolve* over time, and it will be your responsibility to direct that development in a way that supports healthy growth and repair and removes obstacles to that development while making sure that the living system has what it needs to grow: sufficient food, water, love, and protection.

We are living creatures who interact with hundreds and thousands of other living creatures in the complexity of human systems. Simple cause-and-effect explanations for anything break down under these conditions. Instead, we are constantly dealing with another characteristic of living complex adaptive systems—*emergence*—representing the whole that is always something other than the sum of its parts. Life is typified by emergence—even you. When your father contributed one sperm and your mother one egg, they combined to produce someone entirely new and different from any human that lives today or has ever lived—you. Your genes, combined from two parents, then began interacting with a world that has never existed before, and you began interacting with that world. Emergence cannot be determined in advance—it unfolds and evolves constantly. Evolution means change over time that stops only—as far as we know—by death. Living systems keep developing new ways of being throughout time and in interaction with others—the awesome, compelling, and reverential story of life on Earth [74,75].

### 3.4. Imagination: Sociological and Moral

As a trauma-informed leader, you will be called upon to exercise both sociological and moral imagination in a variety of circumstances. Sociological imagination is the idea that you can only understand your own experience and that of your colleagues by understanding the context and history of your and their experiences. This includes an understanding of the history of the organization you are leading [76]. But *moral imagination*? What is that? If leadership is not new to you, you have probably exercised this capacity many times already without giving it a name. Unfortunately, in Western culture, we have inherited the mistaken view that morality is nothing more than a system of universal moral laws or rules that come from the essence of reason and correct moral reasoning just means applying those laws to concrete situations [77]. That’s nice when it works but what happens when there is a lack of clarity, or conflicts with competing ends, or clashes of those values? Those kinds of situations are the ones that you are far more likely to encounter in leading almost any type of organization. That is when you must mobilize your own moral imagination, the crucial process of imaginative moral deliberation by which we are able to explore how experience might play out under the influence of various values and commitments, and circumstances that we must imagine because they have not happened yet [78]. As one writer has noted, “*Imagination is the possibility maker. It is the home of hope and regret*” (loc. 88) [79].

### 3.5. Finding the Right Style

*Leadership is not mobilizing others to solve problems we already know how to solve, but to help them confront problems that have never yet been successfully addressed* (p. 3) [80].

Michael Fullan, 2001, *Leading in a Culture of Change*.

As a trauma-informed leader, you must decide on a leadership style that fits you and the organizational needs at the same time. Living complex adaptive systems *self-organize* which means that order spontaneously arises out of what appears at first to be chaos. Any interference with this process hinders what will naturally emerge and thus interferes with effective function. This capacity for self-organization is why it is not necessary for you to be constantly telling your body what to do. Dr. Cannon was referring to this property when he referred to systems as “biocracy”. When the brain is functioning properly it is complexly interacting with all the other parts of the body helping to balance all of the functions. He declared that *“the body physiologic is a collection of organs, the brain among them, which are interdependent and which, for the welfare of the whole, cooperate. Each one needs the others for perfect function*” (p. 1) [52].

To the great detriment of our organizations, the continuing leadership role that dominates many organizations and societies is that of a top-down authoritarian leadership style. Although authoritarian strategies can be useful in a true emergency, once the emergency has passed such attitudes become an enormous liability because they inhibit the creativity and innovation that self-organization requires and that are necessary for an organization to adapt to change [81,82].

Experience has shown that to sustain a living organization leadership styles need to match the stage of organizational development. Early in development, leaders will need to be purposeful, *authoritative*, clear, and directive. It is unfortunate that the two words—authoritarian and authoritative are so similar because the actual styles denoted are vastly different. History has clearly demonstrated that authoritarians are distant, punitive, controlling, and coercive. Authoritative leaders in contrast, are involved, encourage autonomy in those they lead, expect the assumption of responsibility in others, are consistent, non-punitive, and expect learning and reasoning to occur in everyone [83].

As time passes and the organization develops, leaders must be prepared to loosen the reins so that the organization’s self-organizing capacity can develop properly. This means that leaders must turn toward more democratic, participatory leadership at every level of the organization. As Dr. Cannon pointed out, democracy and biocracy go hand in hand. One scholar has defined the basic structures necessary: “*Democratic leadership is behavior that influences people in a manner consistent with and/or conducive to basic democratic principles and processes, such as self-determination, inclusiveness, equal participation, and deliberation*” [84]. Scholars who have reviewed conceptions of democratic leadership have identified three primary functions: (1) distributing responsibility within the democratic group; (2) empowering the membership; and (3) aiding the democratic group in its deliberations [84]. In most organizations, leadership must be understood broadly since formal and informal leaders and influencers exist at every level. It must never be forgotten that it is the responsibility of leaders to model the fundamental values of the system. They have the power to influence everyone’s attitudes and behaviors in ways that are clearly democratic.

Bogus empowerment happens when leaders talk about sharing power but do not actually do it—it’s just rhetoric [85]. As one scholar has stated, “*We do a terrible job of preparing people to participate in change and of preparing our supervisors to help people participate … We continue to limit workforce participation to relatively trivial issues because we view them as unable to take part in more meaningful discussions. We view participation as a gimmick to increase their satisfaction and motivation, rather than as a potent force to enhance organizational survival”* (p. 43) [86].

Then there is a special problem, typical of nonprofit organizations where a board of trustees are permitted and expected to make key decisions without input from every other part of the organizational body through the narrow communication channel of the CEO, a most undemocratic structure. If such a structure applied to your own body, it would mean that the only information source your body could draw on would be from parts of your conscious mind and every one of your bodily functions would need to be consciously and deliberately regulated. Before long, you would unintentionally make a fatal error, which is exactly what occurs in far too many organizations at present, with chief executives and board members holding the best intentions but being hamstrung by a faulty structure unable to respond to the needs of a complex adaptive living system.

### 3.6. Experiencing Democracy/Biocracy

For several thousand years, humanity has been saddled with antidemocratic organizational structures that are taken as the norm. As a result, there are few truly democratic workplaces and therefore most people have little-to-no experience with practicing democratically and none at all approaching their organizations as biocracies. Such a change requires the development of new skills that can serve as an antidote to the almost universal adaptation that humanity has made to thousands of years of relentless trauma and adversity. Learning to participate in the workplace environment requires an ability to process complex information along with active listening to others. Conflicts inevitably emerge that require the capacity to manage emotions and control impulses that disrupt the group process. Working in participatory environments requires shared decision making and problem-solving that means people must substitute words and reasoning for action. All of this necessitates workers with sophisticated social skills that include the capacity for trustworthy behavior and negotiation, combined with a willingness to compromise and make concessions that promote collective action. Unfortunately, few people have experienced growing up in democratic families or schools, much less workplaces, so all of this may be foreign to them and even frightening; therefore, creating more democratic workplaces will depend upon trustworthy supervision and coaching. One of the pioneers of social, organizational, and applied psychology, Kurt Lewin, pointed out long ago that “Only through practical experience can one learn that peculiar democratic combination of conduct which includes responsibility toward the group, ability to recognize differences of opinion without considering the other person a criminal, and readiness to accept criticism in a matter of fact way while offering criticism with sensitivity for the other person’s feeling” (p. 52) [87].

### 3.7. A Safety Culture

What we call *culture* emerges spontaneously from any human group given sufficient time. Like language and moral intelligence, most human beings are prepared at birth to create culture, beginning with the family. Our genetic inheritance prepares us for culture and then the culture we are in shapes our brain, but there is a constant two-way street as our experiences impact our brain, our genes, *and* our cultures [88]. However, the fact that culture inevitably emerges in a group is no indicator that what emerges serves the interest of individual or group health. In fact, belonging to a culture is often quite the opposite of health, since health requires safety and many group cultures are dangerous places to live and develop, either for some people or for all people, and now even for life itself on the planet.

Organizational culture is the “*pattern of shared basic assumptions that a group has learned as it solved its problems…and that has worked well enough to be considered valid and taught to new members*” (p. 12) [89]. If your vision, as a trauma-informed leader, is of working in and leading a healthy workplace environment, then you must make sure that everyone who enters the organization shares a knowledge base and a language, all of which promotes *alignment*, or getting everyone “on the same page” even though each person’s function in the organization is different. This demands of you a clear sense of moral leadership so that everyone is aligned around purpose, principles, and values and that they are held to those standards [90].

For a biocratic environment to be healthy, everyone must participate in the development and maintenance of a safe environment. This means that part of the organizational structure contains clear definitions of the five key domains of safety that are shared throughout the organization and incorporated into orientation programs, standard operating procedures, organizational policies, and procedures including those directed at employees not just clients. The key domains of safety include physical, psychological, social, moral, and cultural safety, which help to draw respectful boundaries between individuals and groups while defining organizational norms of civil behavior. The maintenance of trust—and the restoration of trusting relationships when trust has been violated—is essential to healthy organizational function [91].

### 3.8. Healthy Social Immune System

We can maintain safety in a world surrounded by microscopic potential invaders such as bacteria and viruses because of our internal security system—our immune system. The immune system is a living subsystem that functions interactively with the other subsystems of our bodies to protect us against hostile invaders and promote repair and recovery when that invasion has not been entirely prevented. Our immune system develops memory, draws upon that memory when a new threat is perceived, and learns from experience—hence the importance of vaccines. Although there are specialists to deal with invasion, the whole body is a part of the immune system, ever watchful and protective. When an invader is perceived, the whole body mobilizes a response—a fever in your body signals that such a response is occurring. When our immune system is overwhelmed, as happened to many people during the COVID-19 pandemic, we become ill or die. If we have not succumbed to the infection, we return to health, and our immune system returns to alert status, ready to fend off the next invasion. However, a wide variety of illnesses and dysfunctions occur when the immune system misidentifies invaders as friends or when it mounts an overwhelming response to a nonlethal threat.

The social immune system is characteristic of our organizational bodies and is defined as the social body’s ability to recognize and respond to threats to its well-being [83]. This means that when the social body is threatened, complex social processes analogous to the way our individual immune system functions are set into motion to defend and protect the organizational body against threat—threat that is in the form of some kind of violence. To be able to respond appropriately, everyone in the environment must shift to a much broader idea of what constitutes violence in a culture.

Because we have lived for many generations within traumatogenic cultures, we only respond to physical violence and expect our “specialists”—law enforcement—to solve the problems. However, every act of physical violence has its origins in the multiple other forms of violence experienced by the violator: psychological, social, moral and cultural, historical and collective forms of violence often beginning in childhood.

Any violation of healthy, nonviolent, and just social norms indicates a breakdown in social immunity. When this occurs the violation must be viewed from a very different and currently atypical perspective recognizing that whoever has committed physical violence is the weakest link in a complex web of interaction that only has culminated in the emergence of physical violence after an unrecognized cascade of previous violations of healthy social norms. As a result, when violence has emerged it is because the entire group has failed to prevent it by failing to respond to and prevent the earlier violations. When we shift our premises in this way, instead of simply chasing after and then punishing the violator, we are compelled to carefully examine how our social immunity broke down. Only in this way can we learn from experience about how to best enhance our own social immune system so it does not happen again. This requires the retention of organizational memory if we are not simply to keep repeating a problematic past.

## 4. The Main Organs of the Organizational Body

Since we are conceiving of the organization as a living being, different roles represent different functions just as there are different functions in the living body that must work together in a wholly integrated way to be healthy. In a social service setting, there are typically four main systems of functions that we will call: Leaders, Clinicians, Direct Service Staff, Indirect Service Staff.

### 4.1. Leadership Team

For our purposes, the human body should be used as the model for the living body of the organization since we all have at least rudimentary knowledge about how our bodies function. The leadership team in that case is the organizational brain and serves the key roles of being the coordinator, regulator, and integrator of multiple functions. As the brain of the organization, the executive team must keep in mind the central mission, purpose, and values of the whole organization since they must coordinate complex adaptations. Complex living systems do not function in isolation, nor can every part be aware of and coordinated with every other part unless there are integrating functions. Our bodies have evolved extraordinary regulatory processes that keep us alive and in balance and that happen automatically. As a trauma-informed leader you need to gather around you a team of people who are on the same page with you from a values-based perspective, but who see things differently enough from you to get a wide variety of input and who together have networked connections with every other part of the organization. Who are the “eyes” of the organization? What happens when something “doesn’t smell right”? Who “speaks” for the organization? These represent some of the sensory functions built into every organization.

The executive team also must play a key role in the digestive system. The digestive system of your body has several jobs to do to keep you alive and functioning. It must turn food into the energy necessary for growth and cell repair, but it must also eliminate waste products. The food necessary for your organizational body to function is money and, once funding has been obtained, your organization must turn that into growth, development, and repair while minimizing waste. Throughout the life of the organization there are many “nutritional demands” that must be met.

Then there is the issue of waste removal. Since energy is needed for growth and repair and must be conserved, we need to be asking three key organizational questions: “What do we already know that we should hold onto?” “What do we need to learn that is new, or forgotten?” “What do we need to get rid of or unlearn?”—the last being the necessary and often unpleasant process of elimination.

We are creatures of habit so unlearning habits, as everyone knows and has experienced, is taxing and beset by repetitive failures long before success. Habits are situated in a part of our brains over which we have little or no conscious control [92]. For organizations to unlearn they must discard old routines in order to make way for new and better ones to be tried [93]. Failure to unlearn useless or destructive strategies is analogous to developing chronic constipation while we reenact failed strategies, hold onto old losses, and become increasingly unable to adapt to changing environmental conditions. Trauma-informed organizations, led by trauma-informed leaders, learn to let go and struggle with the challenge to balance the need to adapt to change with the simultaneous needs to achieve stability, eliminate waste, honor loss, and celebrate successful transitions.

### 4.2. Clinical Team

In living systems, emotions paired with cognitions play a vital regulatory function, drawing our attention toward what is important to survival and keeping us connected to each other while using our rational mind to determine behavior. Part of developing emotional intelligence is honoring empathy—being able to take the perspective of others and experience concern for their wellbeing and desiring to reduce suffering. In caregiving organizations these are primary roles for the clinical team. Think of them as the heart of the organization [94].

### 4.3. Direct Care Staff

The Direct Care staff members function at the interface between the clinical team and the people the organization serves—they are the muscles of the organization. They determine whether the work that needs to be accomplished gets done in a way that serves the overall health of the organizational body. If they do not have clarity about their role and responsibilities and if their knowledge is limited, then organizational function will be profoundly weakened. If they have a clear mission and purpose that serves the healing and recovery of those who have been wounded, those people will get better. Without exercise, muscles atrophy, and if these organizational muscles are not being exercised by new learning, the organizational mission cannot be fulfilled.

### 4.4. Indirect Care Staff

There are still many members of a typical organization who are not being accounted for—IT, Human Relations, Security, Public Relations, Maintenance, Administrative Assistants, Housekeeping—all the people who do their best to keep everyone organized and on track, and in most organizations, there will be some type of oversight board. These are all the Indirect Care Staff. They are the skeletal system of the organization. They provide the support and stability that the muscles need to facilitate movement. They provide protection for all the internal functions of the organization and interface with the external world around them. In the body, the bones create the red and white blood cells that provide the information necessary for health that circulates throughout the body. So too, the Indirect Care staff has a fundamental role in making it possible for information to be carried throughout the entire organizational body.

## 5. The *Creating Presence*™ Implementation Process

The organizing framework described here called *Creating Presence*™, is built around an approach for developing healthier biocratic organizations built on the scientifically grounded and extensive knowledge about trauma and adversity—“*The Science of Suffering*”. The overarching purpose in *Creating Presence*™ is to provide the knowledge base and skill development for collective change starting at the organizational level and gradually impacting every member of the organizational culture through the acquisition of knowledge and experience. It also builds on the landmark work of organizational development theorists Peter Senge and his colleagues who laid out a roadmap for the future workplace as Learning Organizations before the knowledge we now have about the effects of adversity and trauma were widely recognized [95].

In *Creating Presence*™, we have created an online learning approach to helping whole organizations to become trauma-informed and trauma-responsive learning organizations by providing a framework for the emergence of trauma-informed values, knowledge, practice, and skills over the course of at least eighteen months, so that the learning can become deeply embedded in workplace practice without significantly interfering with workplace function. If it takes nine months to create a baby, it takes much longer for an entire organization to reorient itself because it is more than just “training”—it’s about redevelopment.

We use the word P.R.E.S.E.N.C.E. as an acronym for a series of trauma-informed concepts and values that are meant to consistently inform and anchor all personal, interpersonal, and organizational processes. In the implementation process each value is actualized through the acquisition of individual and organizational knowledge, practices, and skills that are tailored to specific needs of the organization and the role of the individual within the organization.

A useful way of thinking about the program is by imagining three interconnected gears of Knowledge, Skills, and Policy/Practice set within the context of relationships that comprise the overall organizational culture and climate. The drivers of the process, the people we are calling the Presence Champions, will be representatives of the various parts of the organizational body, those who have the greatest influence on the organizational culture by virtue of their role/or their personal commitment. According to the National Institute of Implementation the stages of implementation of a complex process like *Creating Presence*™ can be divided into Adoption, Installation, Initial Implementation, and Full Operation [96].

### 5.1. Adoption: The Preparatory Steps

There are several critical administrative tasks that need to involve members of the executive team and that must be completed for an organization to adopt a training program that is going to extend for at least eighteen months. Before universal training can begin, executive team members will need to decide how to make the training itself possible in terms of time, funding, and staffing requirements. Leaders must decide on and roll out an engagement strategy to prepare everyone for what is coming. The people in the organization responsible for information technology will need to be brought onboard since the system is delivered online. The executive team will need to make decisions about who is going to be a part of the Engagement and Enactment Team (E&E Team), which will play a key role in the overall implementation of the process. Once those tasks have been accomplished it is time to plan and implement the administration of the OPTIC assessment tool to all staff. The report generated from OPTIC will be a valuable tool for the Executive team in their own assessment of the needs of their organizational culture.

#### OPTIC (Organizational Presence of a Trauma-Informed Culture)

OPTIC™ is a self-assessment tool in which employees and stakeholders evaluate key practices and characteristics associated with creating a trauma-informed culture that is aligned with the *Creating Presence*™ program [97]. Employees rate their level of agreement on eighty personal (“me” level) and collective (“we” level) domains associated with a trauma-informed culture within an organization, guided through the values embodied in the P.R.E.S.E.N.C.E. acronym. The OPTIC self-assessment tool is an important part of the process and is completed online individually, but responses are then aggregated so that the report formulated describes only the aggregated findings. OPTIC is meant to be used universally before beginning the coursework and after completing the coursework as a guide to organizational strengths and vulnerabilities.

### 5.2. Installation: Engagement and Enactment Team

After the preparatory steps are completed, it is time for the selected members of the E&E Team to begin to meet with the Presence Coach to work through the Introductory Track first and then all the tracks. This group of people (group size will be determined by the organization) will be the Presence Champions who will help the entire organization to complete the coursework by first going through the coursework themselves with a coach who facilitates the process. They will meet with the Presence Coach twice a month for the first nine months and once a month for the second nine months.

This team should be representative of all the different levels and departments of the organization. They will be charged with ongoing agency communication about what is happening in the program, analyzing data and goal setting from OPTIC, helping with training and skill support, role modeling and piloting use of the skills and changes in policy and practice, evaluating current policy and practice to figure out where to focus and collaborate with the coach. Finally, they will be responsible for developing the *Creating Presence*™ *Portfolio*, telling the story of the organizational journey, with the help and support of the coach.

### 5.3. Initial Implementation: Introductory Track for Everyone

Beginning in the seventh month of the program, and after the E&E Team has already worked through the Introductory Track and most of the other Tracks, it is time for the entire organization to begin the training. *Creating Presence*™ starts with an Introductory Track that introduces the objectives for adopting the coursework. It is vitally important that the whole organization is in alignment with the clarity leaders are directing around mission and purpose for the whole staff. In this way we hope to help trauma-responsive organizations to grow and develop.

The next part of the Introductory Track is practical—to share with everyone on the staff how the system works, especially to familiarize them with the different tracks in the learning platform—Leadership, Clinical, Direct Care, Indirect Care—to encourage individuals to decide which track most closely aligns with their own work and clarify the implementation process. The focus then shifts to another important practical issue—creating a safe online learning experience that incorporates ideas around the importance of respecting privacy and enhancing good interpersonal skills.

After those practical steps, the Introductory Track begins introducing the basic knowledge necessary for everyone in a trauma-responsive organization to incorporate into practice, focusing on the human stress response, the problems associated with it, and why collective creativity is so important for organizations given the stress-laden lives we all live. Throughout the course, one of the key ideas is that it is essential that leaders and staff think of their organization as a living body that is a Complex Adaptive System and be prepared to make the paradigm shift that is necessary if significant change is to occur. Along the way the program points out how important it is to stop thinking about organizations as machines and start considering them as orchestras that must be in harmony.

#### 5.3.1. Trauma and Stress in Individuals

The next part of the Introductory Track focuses on stress and trauma as it affects individuals. The problems that arise from exposure to trauma and adversity are frequently complex and interactive and have serious implications for caregiving environments. This is material that is challenging for everyone in an organization to absorb so we have organized the basic information around a set of dual values represented by the acronym. Here we describe why the letters of P.R.E.S.E.N.C.E. help to exemplify many of the problems associated with trauma and their resolution and make connections between the values represented by P.R.E.S.E.N.C.E. and a history of traumatic exposure.

#### 5.3.2. Relationships and Recovery

In the next part of the Introductory Track, we look at the role of relationships and recovery in becoming trauma-responsive and begin with an emphasis on Cultural Safety, the fifth component of safety in any group. Adding cultural safety to physical, psychological, social, and moral safety enables an organization to honor and integrate diversity, equity, and inclusion work as part of an overall trauma-responsive organization.

#### 5.3.3. Trauma and Organizations

The next section of the Introductory Track helps to get everyone on the same page about some basic dynamics of groups and systems. Systems thinking means understanding that, because everyone is connected to each other in a system, for the system to do its work effectively everyone needs to be in alignment with everyone else about basic mission and values, even while each person is doing something different.

#### 5.3.4. Universal Skills

Throughout the entire course, we offer four different kinds of skills to help programs develop resources to meet many of the problems they encounter. We divide these into brain regulation skills, communication skills, group engagement skills, and complexity skills. By the end of the Introductory Track, three vital skills for the entire organization have been introduced and designed to immediately be implemented as “universal precautions”. This term, originally meant to apply to infectious diseases, such as COVID-19, has made us all more familiar with the use of handwashing and facemasks as a way of preventing contagion. Obviously, infectious diseases spread around organizations but so do contagious emotions, which can be disastrous to organizational function. So, in *Creating Presence*™, Presence Meetings, Emotional Management Plans, and Wellness Plans are all universal precautions, necessary for everyone in the organization.

##### Presence Meetings

*Presence Meetings* should be the beginning of every team meeting at every level of the organization including conference calls and Zoom meetings. It is a transition process that helps us move from one highly stressful activity to another. In grounding ourselves for the beginning of a meeting, we increase the level of safety and trust within the organization.

##### Emotional Management Plans

Emotional dysregulation is a primary problem for anyone who experiences chronic stress. It is inevitable that on the job and at home you will at times have difficulty managing your own emotions. Being unable to manage distress can lead to many different problems like taking drugs, impaired job performance, Damaged relationships, escalating interpersonal conflict, smoking, and drinking too much alcohol. Therefore, an Emotional Volume Plan is like wearing a seat belt—everyone needs to use them all the time—another “universal precaution”.

##### Wellness Plans

Because the high incidence of vicarious trauma and burnout can affect the well-being of the entire organization, it is vital that all staff have a defined, flexible wellness plan for self-care that can become part of the supervisory structure as its effectiveness is revisited over time.

### 5.4. The Specialized Tracks: Leadership, Clinical, Direct Service, Indirect Service

The coursework then divides into four separate tracks based on the four typical functional categories of people working in health, mental health, and social service organizations. In each track the coursework is divided into eight modules aligning with the acronym P.R.E.S.E.N.C.E.

The **Leadership Track** addresses the multiple ways that staff in leadership positions can use power responsibly to align attitudes, behavior, and policy with trauma-responsive values and principles, as well as to mitigate the impact of organizational stress on everyone.

The **Clinical Track** is directed at clinically trained personnel and focuses on trauma-specific treatment frameworks, brain regulation tools, and skills for assessing and addressing trauma-related symptoms in the therapeutic relationship. Clinicians also play a vital role in the operations of the organization and are often consulted for advice when interpersonal conflict is apparent. They also are in an excellent position to coach the Direct Support Professionals in areas of clinical knowledge and judgment that are essential for good team treatment.

The **Direct Support Track** provides strategies for constructing a trauma-responsive milieu in which services are delivered as well as offering brain regulation and group engagement tools. There will also be an emphasis on enhancing the interpersonal and relational skills needed to manage trauma-reactive client behavior and promote recovery and growth.

The **Indirect Support Track** will highlight the importance of indirect service providers in shaping organizational culture and providing a positive client experience with a focus on tools for trauma-responsive communication. We have prepared a course for them that allows them to share the basic knowledge that everyone else shares and acquire some basic skills that we hope will help them in their daily function.

### 5.5. The Organizing Acronym for Creating Healthy Culture Using Trauma-Responsive Values

The eight letters of P.R.E.S.E.N.C.E., representing sixteen values, are used as an organizing framework for the coursework and are applied to both an understanding of individuals in health, under stress, and the ways in which the various types of stress can derail people. It is just as important for all staff members to understand what healthy group function looks like, what happens to groups under stress, and how group function easily becomes derailed as a result. Each track has a number of modules, some mandatory and others that are supplemental, that provide specialized material based on each separate role definition, while all staff develop a set of skills for managing difficult situations and promoting organizational health paired with each letter:

− P = Partnership and Power—Promoting shared decision-making
− R = Reverence and Restoration—Promoting respect and healing from the past
− E = Emotional Wisdom and Empathy—Promoting deep understanding and compassion
− S = Safety and Social Responsibility—Promoting safety, self-awareness, and teamwork
− E = Embodiment and Enactment—Promoting insight and empowered behavior
− N = Nature and Nurture—Promoting proactive rather than reactive behavior
− C = Culture and Complexity—Promoting diversity and avoiding oversimplification
− E = Emergence and Evolution—Promoting health growth rather than repetition

### 5.6. Full Operation: Policy and Practice

As the knowledge and skills within the organization expand, there are several organizational issues that as an increasingly trauma-informed leader you may want to focus on based on the findings of the OPTIC tool and the training experiences. Any of these changes can provide you with material to expand your portfolio for certification. Some examples include changes to staff onboarding and orientation, job descriptions and performance evaluations, supervisory policies, performance improvement policies, DEI policies, incident response protocols, organizational safety assessments, strategic planning policies, board/corporate involvement.

Your managers may want to make some important changes in team dynamics, and in managing team conflicts and reenactment dynamics. The clinical drivers of your program may suggest changes to things like client intake protocols, clinical case formulations, trauma assessments, S.E.L.F. psychoeducational groups, restorative work with groups and families, and individual trauma treatment approaches.

### 5.7. Certification and Learning Community

Programs can choose to become certified in *Creating Presence*™. Certification depends on the percentage of employees who complete the coursework and the development of a *Creating Presence*™ *Portfolio* that illustrates the changes made by the organization in adopting the *Creating Presence*™ skills and tells the organizational story. The OPTIC instrument will be administered again at the end of the coursework to give the leadership team an assessment of what work still needs to be done. Organizations can then become members of the *Creating Presence*™ *Learning Community* that will promote the continuing adoption of trauma-informed methods by providing access to online materials that include many other skills and lessons, as well as the shared experiences of other members of the community.

## 6. Discussion

So far, most of the organizations that have been adopting a trauma-informed organizational approach have been social service, mental health, healthcare, and educational programs. Although there are many community-wide and even state-wide efforts to accelerate the process of learning and adaptation, it is yet to be determined how much the trauma-informed knowledge base will impact the business community as a whole and specific sectors individually, and that would be fertile ground for specialists in organizational development research.

The OPTICS tool has not been independently evaluated and that would be a significant and researchable project since each program will be required to use the tool at the beginning and at the end of the educational process. Based on qualitative data from the pilot program for *Creating Presence*™ and the first certified program, as well as other participating organizations, we have already made several significant revisions to the original program. Once we are certain that what we are delivering is achieving our educational goals, we will develop a logic model that can be the framework for obtaining more empirical data about outcomes. Once full implementation is achieved, the program is completely computerized and therefore data collection becomes straightforward. The OPTIC instrument will inform us before beginning the process where the organization’s strengths and vulnerabilities lie, while the track system means that we can accumulate data based on the roles people play in the organization. This then will allow specialized interventions to be developed if necessary for strengthening organizations where they are seen to need the most help.

Finally, the concept of biocratic organizations needs far more exploration. Using knowledge people already have about their own bodies offers entry to an entirely different way of understanding our workplaces and our communities. For example, predatory people spread “moral viruses” throughout the organization, in both for-profit and not-for-profit organizations. These kinds of infections create perfect organizational climates for sexual harassment, bullying, racism, abusive supervision, and other counterproductive workplace behaviors to flourish. Predatory people only have license to pursue their own ends when the “social immune system” of the organization, the community, or the whole society has broken down. More research needs to go into understanding how trauma-organized systems come about and the multiple ways that the media and other social influences promote and support social norms that are predatory as well as how to prevent that from happening and how to create and sustain healthy workplaces from the start.

## 7. Conclusions

Given this brief summary and the claim that all of this represents a “paradigm shift” for everyone, everywhere in how we view past, present, and future conditions, and considering that all these traumatic circumstances have been going on for many thousands of years to every family of the species *Homo sapien sapien*, it becomes much clearer just why human beings have become the most significant disease pathogens to threaten life on Earth [38]. If you are going to take on the responsibility of becoming a truly trauma-informed leader, then you need to *GROK* this. To “grok” is a neologism coined by Robert Heinlein in his 1961 science fiction book, *Stranger in a Strange Land* where the word was a Martian word not easily translated and yet anyone who read the book probably understood what he meant. The Oxford English Dictionary took a crack at defining the word and summarized the meaning as “to understand intuitively or by empathy, to establish rapport with” and “to empathize or communicate sympathetically”. The point for you to understand, as an emerging trauma-informed leader, is that you must truly *grok* this paradigm shift. It is *not* a minor shift. It is not just about some new training on a therapeutic approach, or how to adhere to a governmental policy change. Do not be surprised if you feel like you are a Martian, trying to interpret your language to Earthlings who are frustrated, frightened, and confused by the implications, most of whom will have adapted to adversity and trauma in ways you know nothing about.

We need a peaceful nonviolent methodology—a social revolution—that can only arise out of a new understanding of what we are up against and how we are tightly interconnected with all life on our only planet [98]. We need prototypes for institutions that have incorporated that knowledge into their day-to-day function. The explanations, approaches, and treatments that emerge from this new paradigm are simply more effective at addressing complex human-induced problems. People—and our institutions—can heal and recover, but individual approaches are not sufficient to bring about social change. We need leadership in organizations, communities, and societies to all become trauma-informed before it is too late. The Campaign for Trauma-Informed Policy and Practice (www.ctipp.org, accessed on 31 January 2023) has community groups in every state working toward bringing about trauma-informed change. We hope you will join us.
